# Variability in metagenomic samples from the Puget Sound: Relationship to temporal and anthropogenic impacts

**DOI:** 10.1371/journal.pone.0192412

**Published:** 2018-02-13

**Authors:** James C. Wallace, Jessica E. Youngblood, Jesse A. Port, Alison C. Cullen, Marissa N. Smith, Tomomi Workman, Elaine M. Faustman

**Affiliations:** 1 Department of Environmental and Occupational Health Sciences, University of Washington, Seattle, Washington, United States of America; 2 Environmental Toxicology, Amec Foster Wheeler, Lynnwood, Washington, United States of America; 3 Center for Ocean Solutions, Stanford University, Monterey, California, United States of America; 4 Daniel J. Evans School of Public Affairs, University of Washington, Seattle, Washington, United States of America; Argonne National Laboratory, UNITED STATES

## Abstract

Whole-metagenome sequencing (WMS) has emerged as a powerful tool to assess potential public health risks in marine environments by measuring changes in microbial community structure and function in uncultured bacteria. In addition to monitoring public health risks such as antibiotic resistance determinants, it is essential to measure predictors of microbial variation in order to identify natural versus anthropogenic factors as well as to evaluate reproducibility of metagenomic measurements.This study expands our previous metagenomic characterization of Puget Sound by sampling new nearshore environments including the Duwamish River, an EPA superfund site, and the Hood Canal, an area characterized by highly variable oxygen levels. We also resampled a wastewater treatment plant, nearshore and open ocean sites introducing a longitudinal component measuring seasonal and locational variations and establishing metagenomics sampling reproducibility. Microbial composition from samples collected in the open sound were highly similar within the same season and location across different years, while nearshore samples revealed multi-fold seasonal variation in microbial composition and diversity. Comparisons with recently sequenced predominant marine bacterial genomes helped provide much greater species level taxonomic detail compared to our previous study. Antibiotic resistance determinants and pollution and detoxification indicators largely grouped by location showing minor seasonal differences. Metal resistance, oxidative stress and detoxification systems showed no increase in samples proximal to an EPA superfund site indicating a lack of ecosystem adaptation to anthropogenic impacts. Taxonomic analysis of common sewage influent families showed a surprising similarity between wastewater treatment plant and open sound samples suggesting a low-level but pervasive sewage influent signature in Puget Sound surface waters. Our study shows reproducibility of metagenomic data sampling in multiple Puget Sound locations while establishing baseline measurements of antibiotic resistance determinants, pollution and detoxification systems. Combining seasonal and longitudinal data across these locations provides a foundation for evaluating variation in future studies.

## Introduction

Microorganisms are ubiquitously distributed throughout the earth, creating unique environmental microbiomes that reflect habitat type and conditions [1, 2]. Coastal microbiomes are particularly vulnerable to changing habitat conditions because they are at the interface of land-sea interactions. Increasing urbanization and the changing climate are putting stress on coastal ecosystems [3–5] and have the potential to alter microbiome community structure and function [6–8]. These changes in the microbial community can include increased pathogenicity and antibiotic resistance and have the potential to impact human health and wellbeing [9–12]. In addition to addressing human health concerns, changes in microbial community can serve as early indicators of human impact and environmental degradation [7, 13]. Aquatic environments are particularly relevant because they act as reservoirs for environmental pollutants [4, 10, 14], making the composition of these microbial communities of special importance for understanding the bidirectional relationship between humans and microbes.

In order to fully understand the spectrum of effects caused by anthropogenic contaminants, it is essential to measure baseline microbial community composition in marine habitats. It is important to consider spatial diversity [15] as well as natural temporal variation in microbial community structure and function [16]. Metagenomics is an evolving technology that can be used to characterize microbial community structure and function through sequencing genetic material directly from the environment. Characterization of baseline microbial communities through metagenomics can lead to a deeper understanding of compositional, functional and environmental parameters, as well as trends, that can identify species and functions necessary for sustainable and healthy environments [16, 17].

Metagenomics also allows for the assessment of microbial community diversity and function, integral to the health and stability of marine ecosystems [1]. An extensive meta-analysis of marine communities by Johnston et al. found that anthropogenic contamination of marine habitats was frequently associated with reductions in species richness and/or species evenness (increased dominance of tolerant species) [7]. By exploring these two common diversity measures, it is possible to assess the scope of anthropogenic contamination, a potentially powerful agent of selection acting upon aquatic organisms [18]. In addition to assessing potential diversity loss from anthropogenic contamination, public health implications can be quantified using a broad scale screening tool such as an index to quantify antibiotic resistance determinants in the environment [19]. Currently, environmental health monitoring is centered on culture-based methods of identifying single indicator organisms that have been historically associated with harmful pathogens. Previously, we developed an antibiotic resistance determinant (ARD) index as a decision tool based on metagenomics in combination with next generation sequencing and bioinformatics analysis to survey antibiotic resistance determinants within marine and wastewater treatment plant (WWTP) metagenomes. Characterizing microbial communities by potential anthropogenic impact will allow us to further evaluate adverse pressures and isolate biomarkers capable of assessing human impacts on marine ecosystems and marine impacts on human health.

The Puget Sound estuary is the second largest in the nation and part of an extensive watershed that traverses some of the region’s most populated areas. The waterway provides key economic, recreational and cultural resources for the region, most notably, seafood. Degradation to these environments is detrimental, drawing concern over wastewater and stormwater pollution from system overflows caused by large amounts of annual rainfall. In 2012, approximately 154 million gallons of untreated sewage spilled in Seattle’s local waterways [20]. These contaminated discharges are threatening economic, recreational and coastal uses of the waterways and creating significant health risks for human and aquatic health. Pollution in Puget Sound has become a threat to the shellfish industry. In 2014 over 75 miles of Puget Sound coastline were closed for recreational and commercial shellfish harvest due to concerns over pollution [21]. In a separate incident, high arsenic concentrations in clams resulted in China temporarily banning Washington shellfish imports [22]. These instances demonstrate the economic impact of environmental contamination in the Puget Sound and stress the importance of establishing a robust baseline of ecological parameters to monitor as the city continues to grow and put anthropogenic stress on the ecosystem. Between 2012 and 2013 Seattle was the nation’s fastest growing city [23], highlighting the importance of studying the Puget Sound region during this period of immense growth and change.

In this study we extend our previous metagenomic characterization of Puget Sound by sampling new nearshore environments including the Duwamish River, an EPA superfund site, and the Hood Canal, an area characterized by highly variable oxygen levels. We also resampled a wastewater treatment plant, nearshore and open ocean sites in order to characterize longitudinal and locational variations and establish metagenomics sampling reproducibility.

## Materials and methods

### Sample collection

**Fig 1:**
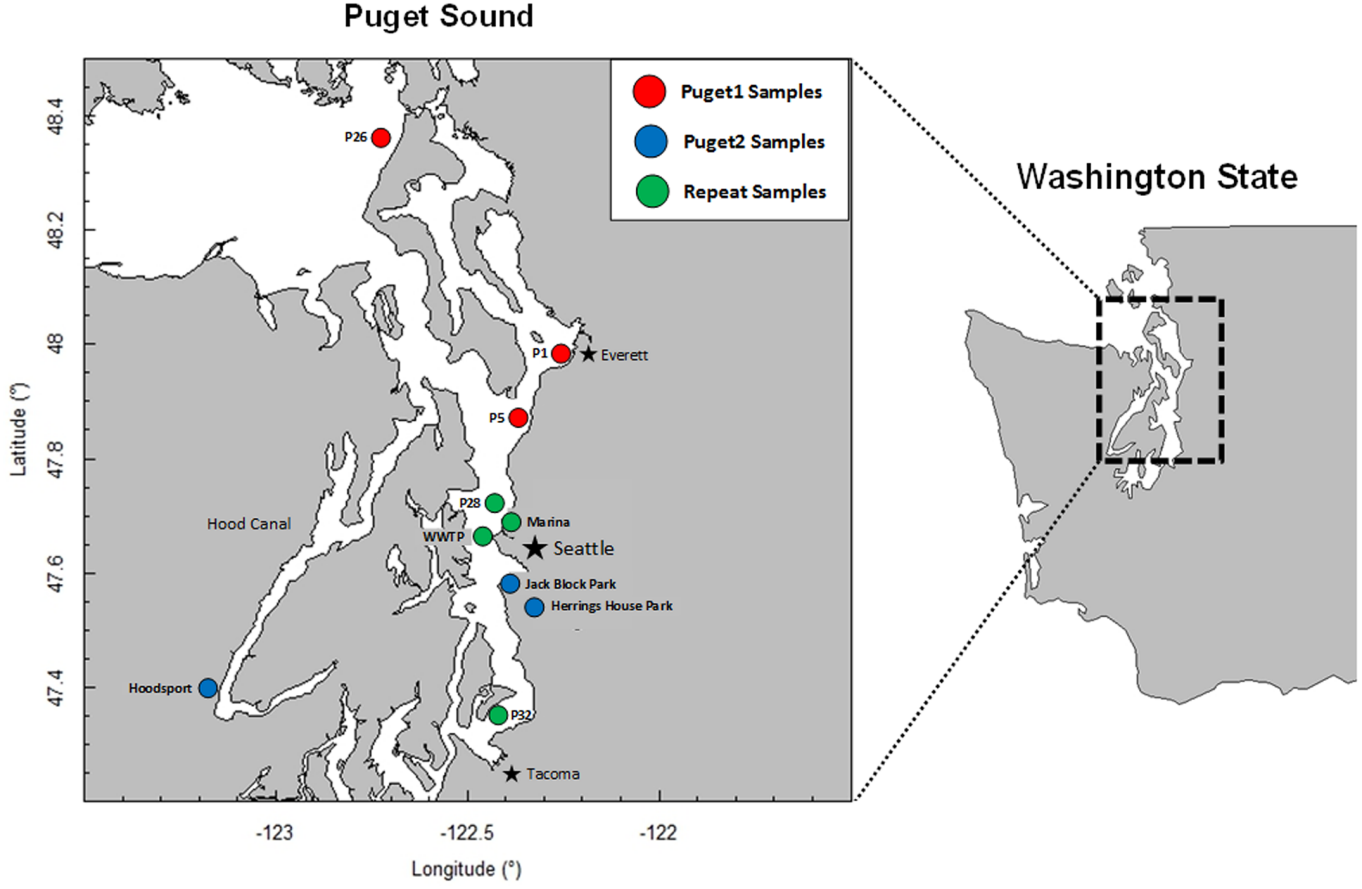
Map of sample sites for Puget1 and Puget2 projects.

This study follows our initial metagenomic survey of Puget Sound by Port et al [24]. That survey (“Puget1”) found unique taxonomic signatures across locations in the Sound as well as an increase in antibiotic resistance gene elements in Marina and wastewater treatment plant (WWTP) samples compared to open sound samples. The current study (“Puget2”) collected additional samples from four of these sites to introduce longitudinal monitoring of Puget Sound and evaluate environmental health concerns for highly impacted areas of the Puget Sound. These sites were selected to coincide with previous sample collection sites including water samples collected from three aquatic environments located throughout the Puget Sound watershed and one effluent sample from a WWTP in Seattle, Washington. To these sites we added three new sampling sites in nearshore environments. The Hood Canal sample was taken from a site proximal to Hoodsport, Washington which is characterized by highly variable oxygen levels. Two Duwamish River samples from Herrings House Park (HHP-2) and Jack Block Park (JBP-2) were collected. The HHP-2 sample was collected from an Environmental Protection Agency superfund site with a history of over 100 years of industrial and urban use pollution. The JBP-2 site is situated at the mouth of the Duwamish River downstream from HHP-2. Duwamish River contaminants include PCBs (polychlorinated biphenyls), PAHs (polycyclic aromatic hydrocarbons), arsenic, dioxins and furans [25]. Fig 1 and Table 1 show map locations and metadata for 11 metagenomic samples from Puget1 and Puget2 projects used in this study.

**Table 1:** Environmental metadata for 11metagenomic samples.

Project	Sample	Abbrev.	Location	Latitude (N)	Longitude (W)	Collected	Depth [M]	Temp. [°C]	Salinity [PSU]	Predicted proteins
Puget2	Herring's House Park	HHP-2	Nearshore	45.561024	-122.350121	7/10/2012	5.0	15.21	14.97	395,414
Puget2	Jack Block Park	JBP-2	Nearshore	47.584796	-122.368733	7/9/2012	5.0	13.44	25.04	127,416
Puget2	Hoodsport	Hoodsport-2	Nearshore	47.403256	-123.141155	10/13/2011	5.0	18.85	22.61	170,561
Puget1	Marina	Marina-1	Nearshore	47.686743	-122.403775	12/20/2010	0.5	8.6	18.7	224,827
Puget2	Marina	Marina-2	Nearshore	47.686743	-122.403775	7/12/2012	5.0	15.0	23.4	234,218
Puget1	WWTP	WWTP-1	WWTP	47.661667	-122.433333	1/31/2011	N/A	14.9	<0.5	121,386
Puget2	WWTP	WWTP-2	WWTP	47.661667	-122.433333	8/1/2012	N/A	20.3	0.4	144,936
Puget1	P28	P28-1	Open Sound	47.700556	-122.450556	10/29/2010	5.0	11.5	32.2	152,756
Puget2	P28	P28-2	Open Sound	47.700556	-122.450556	10/10/2011	5.0	11.9	30.0	134,311
Puget1	P32	P32-1	Open Sound	47.333333	-122.434722	10/30/2010	5.0	11.5	30.2	142,611
Puget2	P32	P32-2	Open Sound	47.333333	-122.434722	10/10/2011	5.0	12.4	29.3	135,538

Puget2 open sound samples from stations P28 and P32 were collected during a University of Washington research cruise aboard the R/V Thomas Thompson from October 10–11, 2011. Shilshole Marina, Duwamish River, Hoodsport and WWTP effluent samples from the King County West Point wastewater treatment plant in Seattle, Washington, were collected in July, 2012. Puget1 study open sound samples from stations P28 and P32 were collected from October 29–30, 2010. Puget1 Shilshole Marina and WWTP effluent samples were collected December 20, 2010 and January 31, 2011 respectively (Table 1). At each sampling location except the WWTP effluent site, 80-liters of surface water were collected using a Niskin bottle rosette mounted on a conductivity-temperature-depth (CTD) sensor array from approximately 2 to 5 meters of depth. The WWTP samples were collected by siphoning treated water directly from the effluent line inside the WWTP. Water samples were stored in carboys at ambient temperature until filtration. Physical and chemical parameters were measured using a multi-parameter water quality meter (Hanna Instruments, HI9829-01/4T) to measure temperature, salinity, dissolved oxygen, pH, turbidity, and total dissolved solids (S1 Table). For the cruise samples P28 and P32, physicochemical conditions including temperature, salinity, and dissolved oxygen levels were measured using a CTD sensor array mounted on a Niskin bottle rosette. No permissions were required for samples collected from the open sound, Shilshole Marina, Duwamish River and Hoodpsort sites as these are all public areas. West Point WWTP provided permission for the WWPT effluent samples.

#### Filtration

Surface water from each location and the WWTP samples were pumped through a single-channel peristaltic pump system (Cole-Palmer, U.S.A.) and sequentially fraction filtered through a 3-μm (147 mm) polycarbonate membrane (Sterlitech, WA) and a 0.2-μm (147 mm) Supor membrane (PALL Corporation, U.S.A.) to obtain the bacterial fraction. Cruise samples were filtered on board immediately after water collection while the other samples were transported back to the lab and filtered within two hours of collection. Depending on the turbidity at a sampling location, approximately 6 to 40 liters of water were filtered per set of membranes. Filters were preserved in a sucrose lysis buffer (50 mMTris HCl, 40 mMEDTA, and 0.75 M sucrose), cut in half and stored at -80°C for downstream DNA extraction.

#### DNA extraction and sequencing

Half-filters were thawed and cut into fourths. For each sample, total metagenomic DNA was isolated and extracted from a quarter filter using the PowerWater^®^DNA Isolation Kit (Mo Bio Laboratories, CA). Protocol modifications included: a 10 min water incubation at 65°C after the addition of Solution P1 (alternate lysis method) and a lysozyme digestion (100mg/ml) at 37°C for 45 minutes followed by a RNase digestion (100mg/ml) at 37°C for 15 minutes prior to the first centrifuge. DNA concentrations were quantified using the Quant-iT Picogreen dsDNA Assay Kit (Invitrogen, U.S.A.) and ranged from 81 to 157 ng/μl per sample. Gel electrophoresis confirmed high molecular weight DNA fragments. Total DNA was submitted for pyrosequencing on a Roche/454 GS FLX Titanium system in the Department of Microbiology (University of Washington).

#### DNA sequence processing and quality control

Roche 454 sequence reads were trimmed to remove barcodes and secondary adapter sequences and reads that contained more than 5% of any one nucleotide. Raw reads were assembled into contigs using Newbler v. 2.6 (Roche Diagnostics-454 Life Sciences) using a 95% minimum overlap identity and default settings.13,953 contigs greater than 500 base pairs in length were assembled for all Puget2 projects totaling 16.4 million base pairs with an N50 of 1,300 base pairs compared to an N50 of 1,100 nucleotides in our previous study. Additional filtering of sequence reads was performed using the Metagenomics Rapid Annotations based on Subsystems technology (MG-RAST) server version 3.3.3.3 [26]. Low quality sequences were removed during unassembled sequence upload using MG-RAST omitting any reads with a read length greater than two standard deviations from the mean sample read length or reads containing greater than five ambiguous base pairs. In addition, the MG-RAST de-replication option was selected to remove artificial replicate sequences produced by sequencing artifacts retaining a single representative sequence from a duplicate cluster [27]. Overall, high-throughput sequencing for open sound, nearshore and WWTP effluent DNA generated a total of 559,995 quality-filtered sequence reads totaling 245,175,190 base pairs with a mean read length of 437 base pairs that were used for downstream analysis.

#### Metagenome sequence accession

Sequence data for the 2010 Puget1 dataset is available through the MG-RAST server (http://metagenomics.anl.gov) under the MG-RAST identification numbers 4460180.3 (P28-1), 4460182.3 (P32-1), 4460189.3 (Marina-1) and 4460190.3 (WWTP-1). The 2012 Puget2 dataset is available under the MG-RAST identification numbers: 4517543.3 (P28-2), 4517544.3 (P32-2), 4517545.3 (Marina-2), 4517546.3 (WWTP-2), 4517540.3 (HHP-2), 4517542.3 (JBP-2) and 4517541.3 (Hoodsport-2).

### Data analysis

#### Microbial community composition classification

DNA sequences for all samples were translated into predicted protein sequences using MG-RAST. Protein sequences were searched using DIAMOND BLASTP [28] and the May 2017 NCBI non-redundant protein database (122,307,828 sequences) using default DIAMOND settings. Diamond format files.DAA were imported into MEGAN Community Edition version 6.6.7 [29] and assigned to taxonomic ranks using default MEGAN LCA (Lowest Common Ancestor) settings. An overview of the analysis framework is shown in S1 Fig.

#### Statistical analysis

Statistical analysis was conducted using STAMP software [30]. Two-sample comparisons were evaluated using Fisher’s Exact Test and Benjamini-Hochberg multiple test correction. All results reported as significant had a p-value < .05. All MEGAN individual bacterial phylum, family and species counts were normalized to the total number of MEGAN assigned counts for each sample. Heat maps for multiple samples were generated by STAMP software using ANOVA sorting by rows and columns with an average neighbor (UPGMA) clustering threshold of .75. Linear regression analysis (S2 and S3 Figs) was calculated using JMP software version 13 (SAS Institute Inc., Cary, NC).

#### Species diversity

Alpha diversity was calculated for each sample’s species counts using the Shannon diversity index (*H*) [31] using the VEGAN R package [32]. Adjusted species were calculated using the formula exp (*H*). Beta diversity was computed by MEGAN using a square root Jensen-Shannon divergence matrix.

#### Analysis of antibiotic resistance determinants and human impact indicators

Antibiotic resistance determinants (ARDS) including antibiotic resistance (AR) genes, mobile genetic elements (MGEs) and transcriptional regulators (TRs) and human impact indicators (PAHs and detoxification systems) were measured using hidden Markov model (HMM) analysis. HMMSEARCH version 3.1 was used to search Pfam version 29.0 [33] and ResFam version 1.2 [34] databases to identify ARDs, MGEs and TRs as previously described [35] using the “trusted cutoff”. HMMs derived from polycyclic aromatic hydrocarbon pathway genes [36] were searched as indicators of PAH contamination using an e-value < 10^−5^. The lower Duwamish waterway (where HHP2 and JBP2 are located) is an EPA superfund site heavily contaminated with PAHs, PCBs, arsenic, dioxins and furans. HMMs derived from marine detoxification system genes included transporters, oxidative stress and metal resistance genes [37]. Pfam percentages were normalized to the total number of Pfams identified per sample.

Human pathogens were measured by comparing 46 genera from the Microbial Rosetta Stone Database [38] to MEGAN genus rank assignments. Sewage associated bacterial families [39] were compared to MEGAN family rank assignments.

## Results and discussion

Proteobacteria are found in every environment and include the largest and most diverse division among prokaryotes and the majority of agricultural, industrial and medical relevant organisms [40]. As shown in S2 Table, Puget Sound metagenomic microbial communities were predominantly composed of the phylum Proteobacteria, ranging from approximately 47% in the freshwater WWTP-1 effluent to over 67% in in the JBP-2 sample at the mouth of the Duwamish River. The two next most abundant phyla were Bacteroidetes (38%) in the Duwamish River HHP-2 sample and Actinobacteria (28%) in the WWTP-2 sample. The phylum Firmicutes, which contains human pathogenic genera such as *Streptococcus*, was found mainly in WWTP samples (3.3%-7.8%) and at less than 0.5% abundance in all other samples.

**Fig 2:**
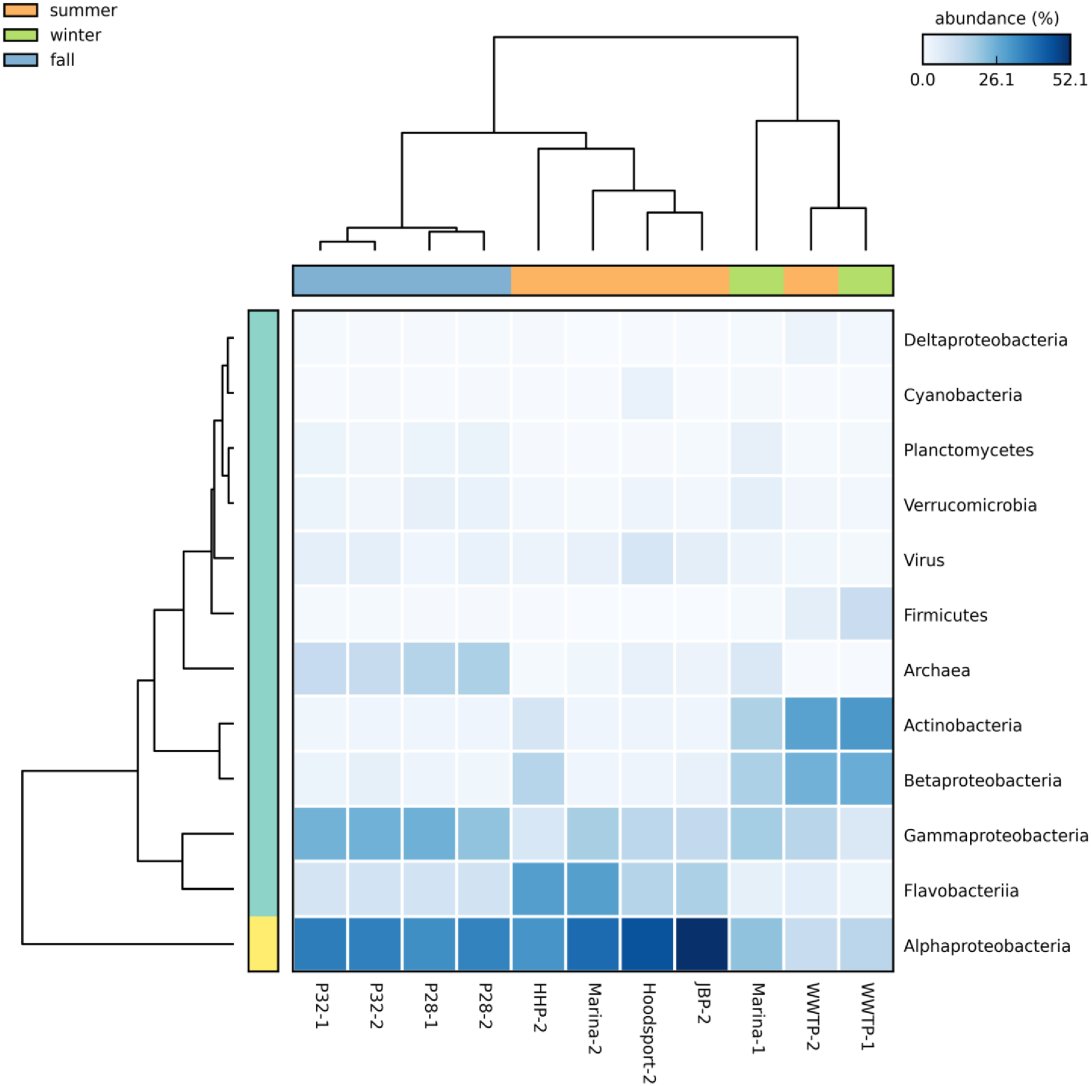
Regional and seasonal community composition differences in Puget Sound showing relative taxonomic abundances for 11 metagenomic samples. Increasing taxonomic abundance is indicated by darker blue boxes. The horizontal color bar at the top reflects season of sample collection. Seattle Shilshole marina nearshore samples (Marina-1 and Marina-2) showed multi-fold seasonal differences between winter and summer measurements in Actinobacteria, Betaproteobacteria, Flavobacteriia and Alphaproteobacteria classes. Archaea were most prominent in offshore P28 and P32 samples and comprised as much as 17% (P28-2) in relative abundance.

WWTP and nearshore Marina sites were sampled in different seasons and P28 and P32 open sound sites were sampled in the same season in different years. As shown in Fig 2, open sound samples P28 and P32 clustered together when resampled with at most a 3% difference in relative proportion of P28 Proteobacteria abundance. Alternatively, the Marina samples showed the largest differences in microbial community composition across samples taken in different years and different seasons as the phylum Bacteroidetes increased over four-fold in relative abundance from winter to summer seasons while Actinobacteria fell from over 20% to less than 3%. Our previous Puget1 study revealed a strong negative correlation between Actinobacteria abundance and salinity. Both Actinobacteria and Betaproteobacteria showed a strong negative relationship between abundance and salinity and Alphaproteobacteria a positive relationship when evaluating combined Puget1 and Puget2 data (S2 Fig). The association between salinity and Alphaproteobacteria and Betaproteobacteria abundance was consistent with previous research evaluating salinity gradients in temperate estuaries [41].

WWTP samples showed increases in Proteobacteria from winter to summer and decreases in Firmacutes. Archaea were most prominent in offshore P28 and P32 samples collected a year apart in relative abundance.

### Alpha diversity

The diversity of marine ecosystems is integral to their stability and function [7]. Alpha diversity measures species diversity within a metagenomics sample. The Shannon Diversity index describes the alpha diversity of the community as both a function of abundance and evenness. As shown in Table 2, Shannon diversity ranged from a high of 5.2 down to 2.8 with the Marina summer 2012 sample showing the least diversity with 17 equally-common species and the WWTP 2012 effluent sample showing the most diversity with 194 equally-common species. Open sound sample sites in Puget Sound showed small differences in diversity and the adjusted number of species, whether in the same or different seasons or locations. However, Marina samples showed a dramatic decrease in diversity between winter and summer samples and WWTP samples showed a large increase in diversity. Although seasonal changes in diversity measures observed in the Marina samples might have been driven by environmental factors, a recent global study found no correlation between alpha diversity and salinity while diversity increased with increased temperatures [42]. Repeat open sound samples, which were taken under similar environmental conditions exhibited highly similar species level composition. The second lowest Shannon diversity was measured in the Hoodsport sample, a region subject to low dissolved oxygen (DO) levels. Previously, alpha diversity has been shown to be negatively associated with DO in this region [43] which may reflect the high DO concentrations measured in this sample.

**Table 2:** Alpha-diversity for 11 metagenomic samples.

Project	Sample	Abbreviation	Location	Shannon Diversity H	Adjusted Species exp(H)
Puget2	WWTP	WWTP-2	WWTP	5.27	194.9
Puget1	WWTP	WWTP-1	WWTP	5.07	159.5
Puget1	Marina	Marina-1	Nearshore	4.26	70.9
Puget2	P32	P32-2	Open Sound	3.62	37.4
Puget2	P32	P32-1	Open Sound	3.61	37.1
Puget1	P28	P28-2	Open Sound	3.6	36.8
Puget2	P28	P28-1	Open Sound	3.58	35.7
Puget2	Herring's House Park	HHP-2	Nearshore	3.57	35.7
Puget2	Hoodsport	Hoodsport-2	Nearshore	3	22
Puget2	Jack Block Park	JBP-2	Nearshore	3.09	20.2
Puget2	Marina	Marina-2	Nearshore	2.86	17.5

### Beta diversity

**Fig 3:**
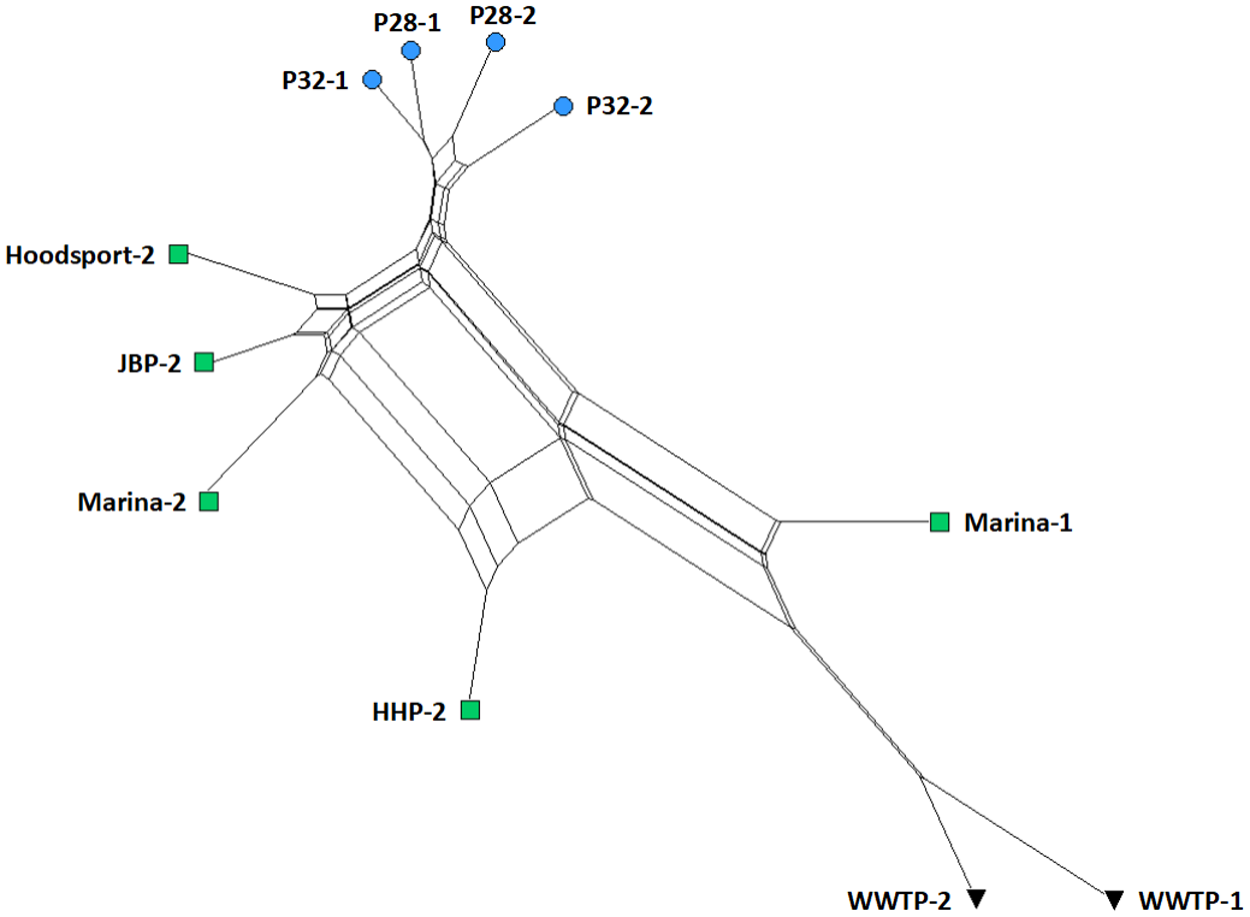
Class rank beta-diversity for 11 Puget Sound samples. Nearshore marina samples showed the largest phylogenetic distance for repeat samples in different seasons.

Beta diversity measures differences between different environments by quantifying their shared or dissimilar taxa [44]. Fig 3 highlights beta diversity for 11 Puget Sound samples at the taxonomic class rank using Jensen-Shannon divergence clustering and a neighbor-net method unrooted phylogenetic network. Puget1 and Puget2 samples clustered together in three main groups: (1) Wastewater treatment plant (WWTP); (2) Nearshore; and (3) Open sound samples. Marina summer and winter samples showed the largest distance between samples from the same location sampled in different seasons.

### Seasonal variation in dominant microbial species

Our previous Puget Sound WMS study [24] identified the predominant microbial species in six open sound and Marina samples as a member of the Roseobacter clade and most similar to *Rhodobacterales HTCC2255* [45]. DNA recruitment plots showed a seasonal increase in nearshore Marina DNA sequence reads mapping to *Rhodobacterales HTCC2255* between winter and summer samples (S4 Fig). The Rhodobacterales are surface-colonizing bacteria that play important roles in marine fisheries and ecosystems [46]. Other members of the Roseobacter clade such as *Planktomarina temperata* isolated during spring phytoplankton blooms are aerobic anoxygenic phototrophs able to harvest light as an energy source [47]. Hence, *Rhodobacterales HTCC2255* abundance may be impacted differences in daylight between summer and winter seasons.

**Fig 4:**
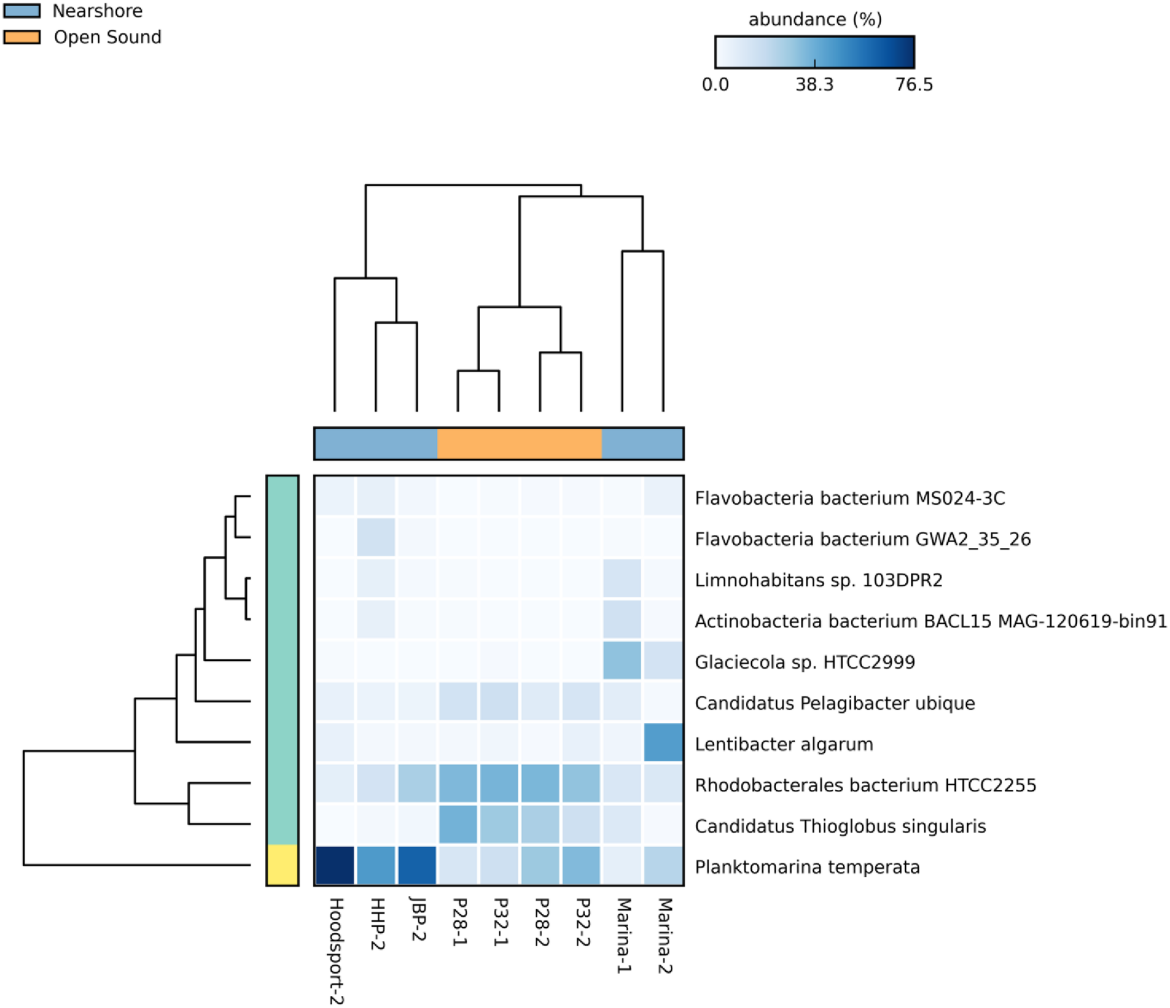
Relative abundances for the 10 most common bacterial species in 5 nearshore and 4 open sound samples. Increasing taxonomic abundance is indicated by darker blue boxes. Nearshore samples (blue bar) clustered by sampling season and open sound samples (orange bar) clustered by sampling location over different sampling years.

Following our original Puget1 report, additional highly abundant bacterioplankton species have been isolated and sequenced from marine environments providing greater taxonomic resolution at the species level. Some dominant species were common to both nearshore and offshore sites while others were found almost exclusively at individual sample sites. Fig 4 shows the distribution of the 10 most common bacterioplankton identified by MEGAN at the species taxonomic rank.

Dominant microbial species common to both nearshore and open sound locations included the Alphaproteobacteria *Planktomarina temperata* and *Rhodobacterales bacterium HTCC2255*. *P*. *temperata* showed the greatest abundance in nearshore samples with a ten-fold increase in the Marina summer sample possibly reflecting the impact of seasonal sunlight availability.

Recently, the Alphaproteobacterium *Lentibacter algarum* was isolated from coastal Chinese waters [48] and its genome was sequenced. This bacterium greatly surpassed *Rhodobacterales HTCC2255* (S4 Fig) as the most abundant species in Marina winter and summer samples which showed large seasonal differences. *L*. *algarum* represented 10% of all taxonomic assignments and 30% of assignments at the species rank for the Marina summer sample representing a twenty-fold difference in abundance compared to the next closest sample. Overall, marine *Rhodobacteraceae* such as *L*. *algarum* comprise as much as 30% of phytoplankton in pelagic environments [49]. The ability to quantify other species rank abundance will increase as more *Rhodobacteraceae* genomes become available in databases which could supplant *L*. *algarum* as the dominant species in Marina samples. Another species, the Gammaproteobacterium *Glaciecola sp*. *HTCC2999*, was also found almost exclusively in Marina winter and summer samples. *Glaceola* species abundance has been associated with diatoms during spring phytoplankton blooms [50].

*Flavobacteria* genomes in the phylum Bacteroidetes were included among the dominant 10 bacterial species. *Flavobacterium MS0243-C*, poorly represented in cultures derived from environmental samples, is abundant along with other closely related species in North Atlantic coastal waters and features a small (< 2.4mb), streamlined genome [51]. Recent WMS sampling in the Gulf of Maine detected a similar pattern of seasonality in *Flavobacteria* species abundance [52]. *Flavobacteria bacterium GWA2_35_26* [53] was recently isolated and sequenced from an aquifer near the Colorado River and was present almost exclusively in the Herring’s House Park Duwamish River sample. Although the Flavobacteriia class showed a multi-fold seasonal difference in Marina samples (Fig 2), neither of the above *Flavobacteria* species played a prominent role suggesting that important marine *Flavobacteria* species remained unidentified. As in our previous Puget1 study, we saw a strong association between salinity and abundance in the most common bacterial classes. However, the large increase in Flavobacteriia class abundance was not associated with salinity (S2 Fig). Additionally, although others have shown that the global distribution of predominant marine bacterioplankton is most closely correlated with temperature and latitude [54] we found only a weak association between temperature and class abundance in the gamma-proteobacteria and no significant association in other predominant classes (S3 Fig). For latitude, *L*. *algarum*, the predominant bacterial species found in the marina-2 sample, was originally isolated in Qingdao, China with a latitude approximately 800 miles south of the Seattle, Washington sample site.

### Taxonomic and functional analysis of potential anthropogenic determinants related to public health

#### Sewage associated bacterial families

**Fig 5:**
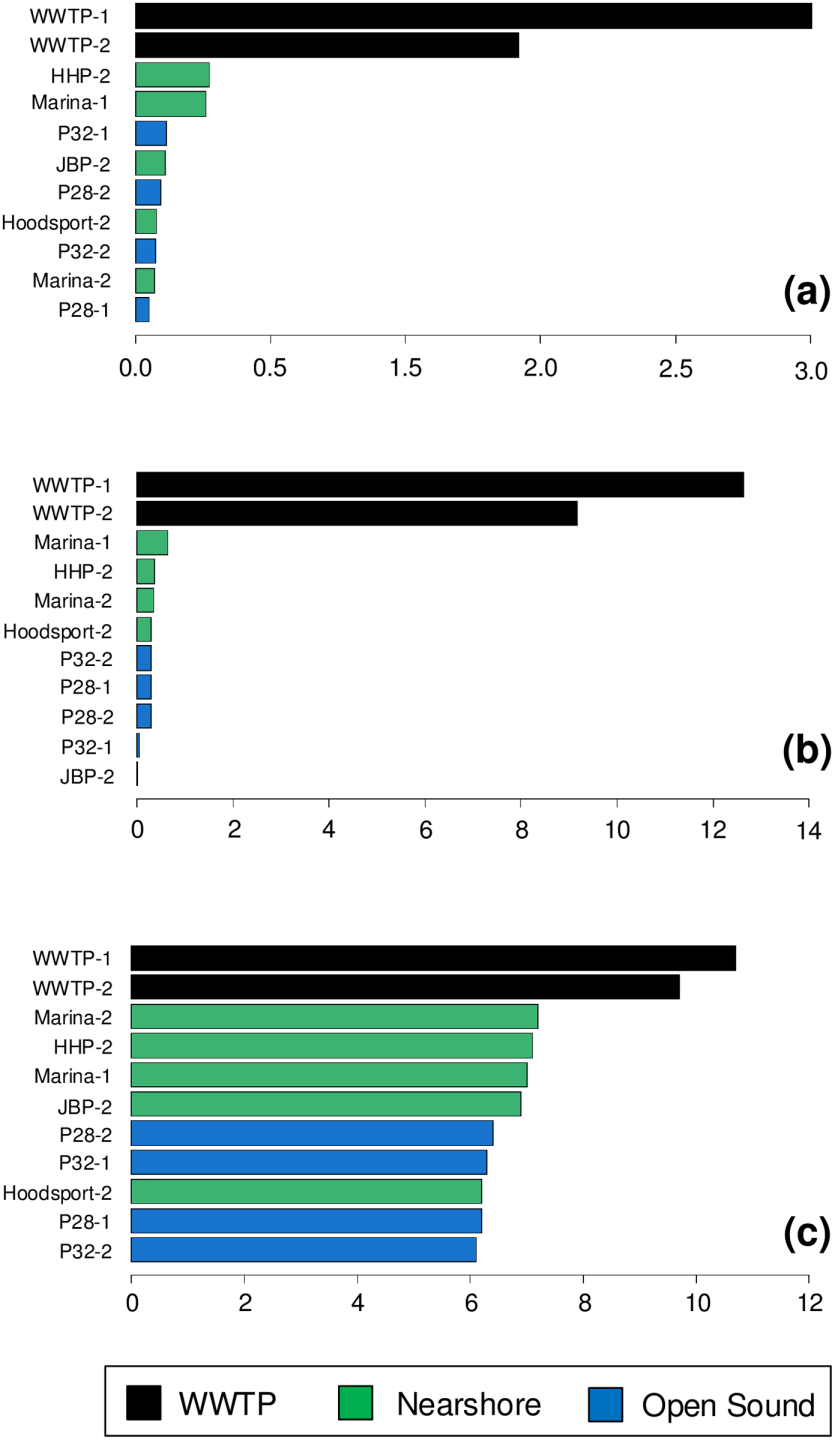
Potential anthropogenic determinants and detoxification systems related to public health. (a) Percent of MEGAN LCA family taxonomic rank assignments for sewage influent bacterial families. (b) Percent of MEGAN taxonomic assignments for 28 human bacterial pathogenic genera. (c) Combined percentage of reads assigned to antibiotic resistance determinant elements, polycyclic aromatic hydrocarbon (PAH) degradation and detoxification systems Hidden Markov models.

MEGAN LCA family taxonomic rank assignments for sewage associated bacterial families [37] showed large seasonal differences in wastewater treatment plant and marina samples (Fig 5a) from winter to summer. The Lower Duwamish sample (HHP-2) from Herring’s House Park showed small but significant differences in sewage associated families compared to the mouth of the Duwamish River at Jack Block Park (JBP-2).

The largest seasonal differences were detected in wastewater treatment plant effluent and marina samples. Significant differences were also detected between the upstream Duwamish River sample from Herring’s House Park (HHP-2) and the mouth of the Duwamish River at Jack Block Park (JBH-2).

**Fig 6:**
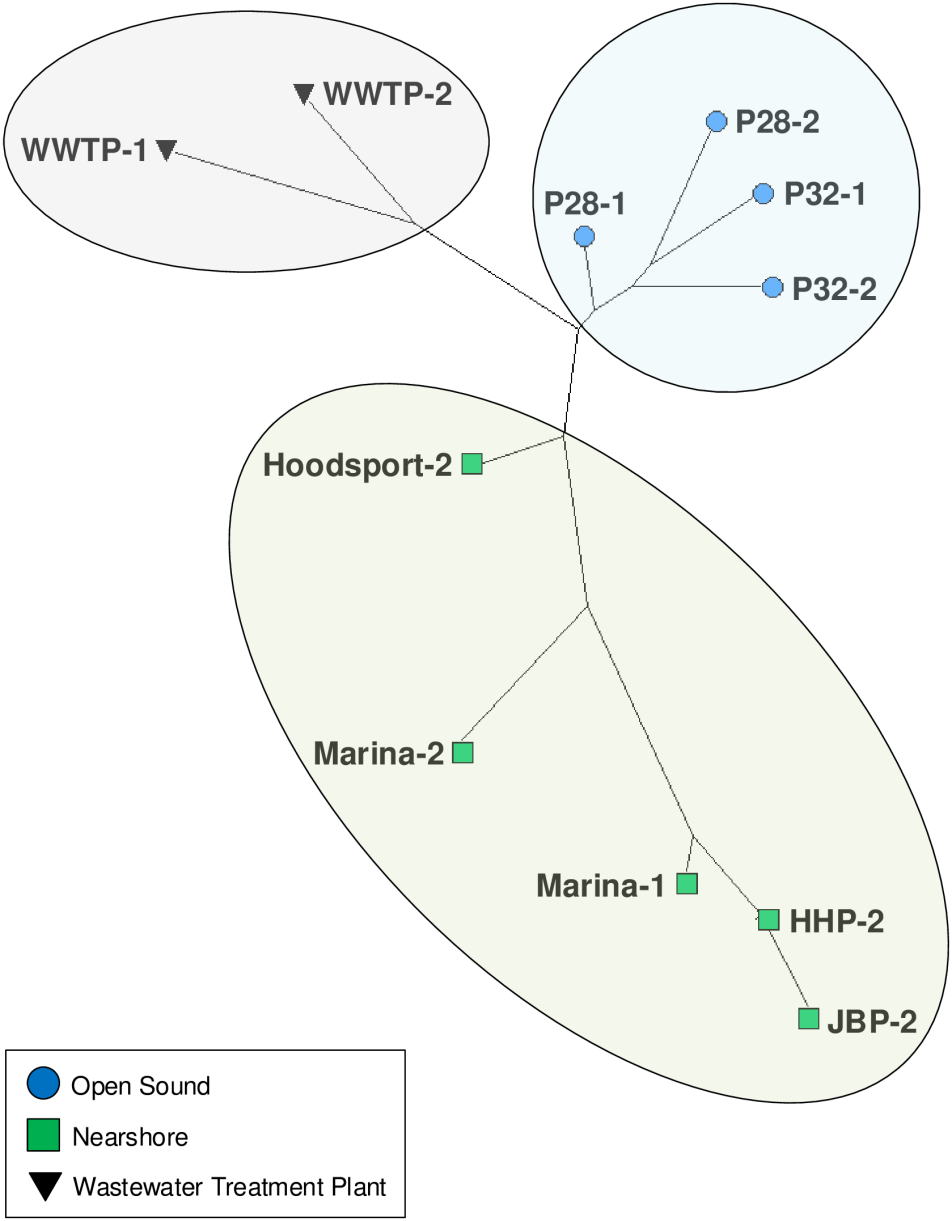
Neighbor-joining tree representation of phylogenetic distances for sewage associated bacterial families. Open sound samples were taxonomically closer in relative abundance to WWTP samples than Marina and Herring’s House Park samples although smaller in absolute abundance as shown in Fig 5a.

Fig 6 shows a neighbor-joining tree representation of phylogenetic distances for sewage associated bacterial families based on relative abundances. Interestingly, open sound samples were more taxonomically similar to WWTP effluent samples than nearshore samples proximal to the WWTP while much less in absolute abundance shown in Fig 5a. This may indicate a low-level but pervasive sewage influent signature in Puget Sound surface waters.

#### Pathogenic bacteria

**Fig 7:**
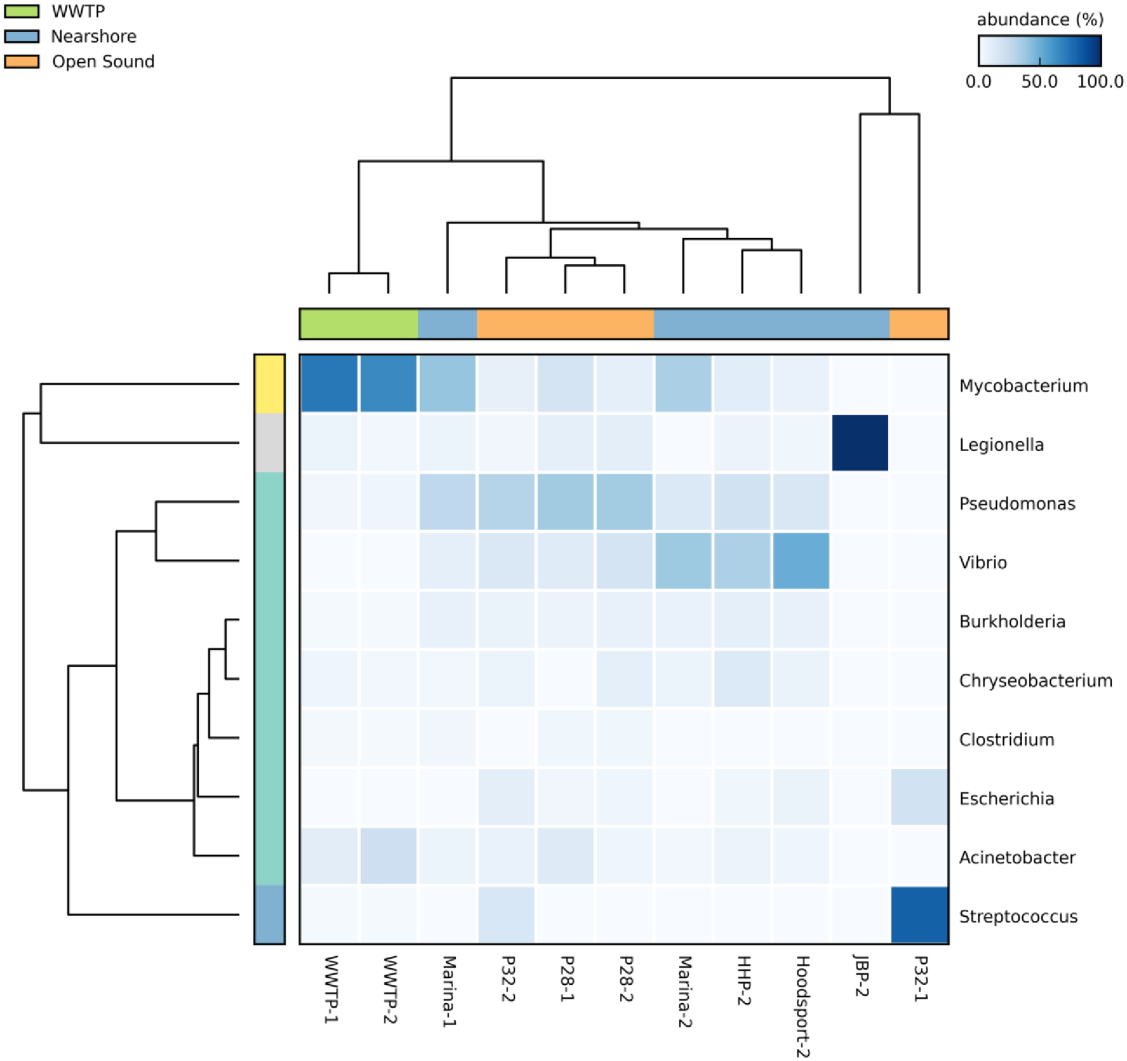
Relative abundances of top 10 pathogenic genera for 11 metagenomic samples. Increasing taxonomic abundance is indicated by darker blue boxes. The horizontal color bar at the top reflects sample collection location (WWTP, nearshore or open sound samples).

As in our previous study, the greatest number of human pathogenic genera were found in WWTP effluent samples where 27 were detected out of a total of 28 pathogens found among all samples. Pathogenic genera showed the greatest increase in summer (approximately 4%) versus winter WWTP effluent samples in assigned protein sequences. Fig 5b shows combined percentages of human pathogens mapped at the genus level for 11 metagenomic samples. *Mycobacteria* were the most common pathogenic bacteria genera detected in WWTP effluent in both winter and summer samples. Coincidentally, *Mycobacteria* were also the most common pathogen related genus in both the winter and summer Marina samples whose site is closest in proximity to the Seattle West Point WWTP shown in Fig 1. Fig 7 shows relative abundances for the top 10 genus rank pathogens.

At the species rank, *E*. *coli* was detected in all sample sites and samples except for the Marina-1 winter sample. *Vibrio parahaemolyticus*, a naturally occurring enterotoxic bacterium causing acute gastroenteritis in humans consuming raw shellfish [55], was detected exclusively in the Hoodsport-2 nearshore sample.

*Streptococcus suis* colonizes the upper respiratory tract of adult pigs and can be transmitted to humans where it can lead to serious infections including sepsis and meningitis. *S*. *suis* was detected in all four WWTP and P32 samples.

*Flavobacterium branchiophilum*, the causative agent of bacterial gill disease affecting cultured freshwater fish species (50), was found exclusively in the Herring’s House Park Duwamish river sample (HHP-2).

#### Variation in antibiotic resistance determinants and detoxification systems

**Fig 8:**
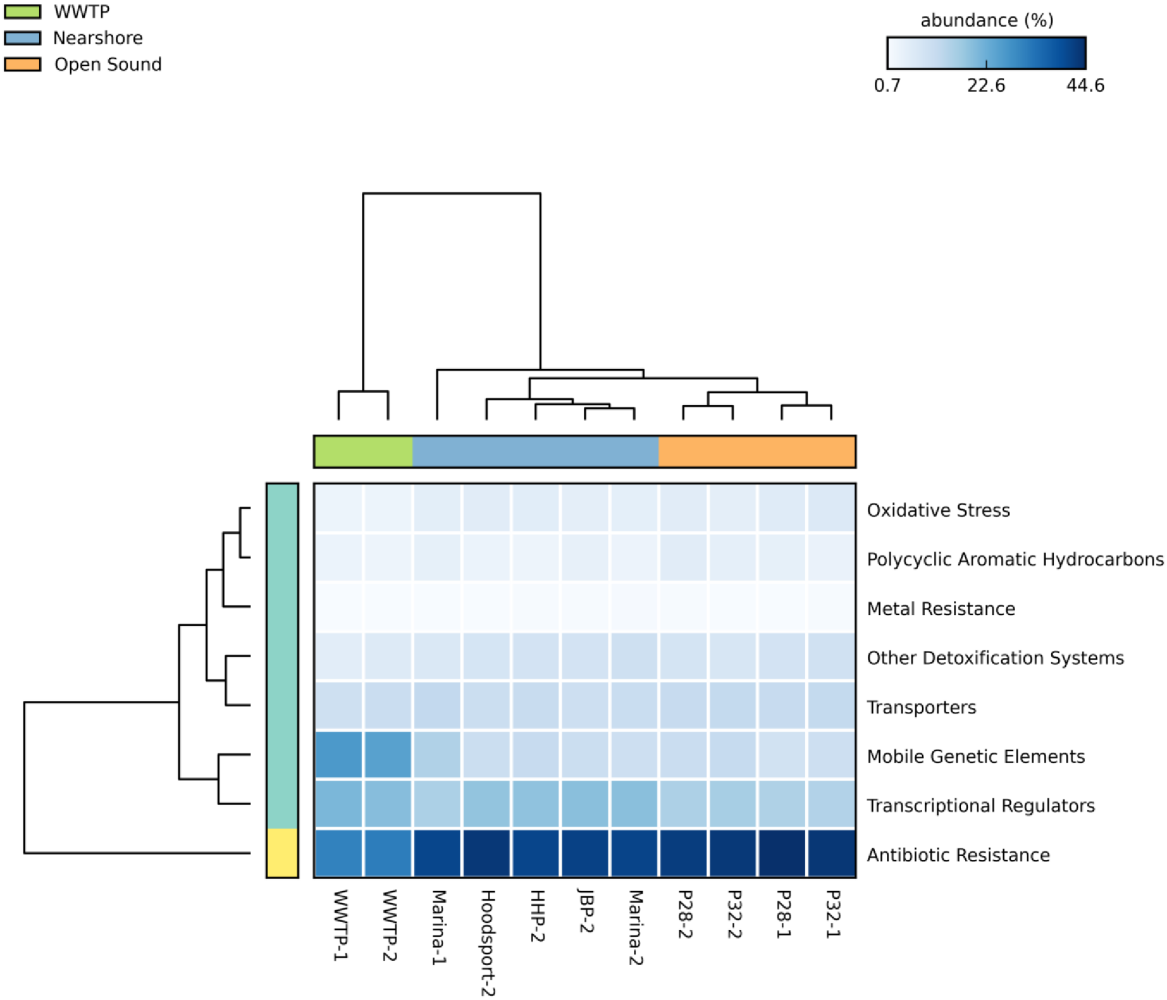
Antibiotic resistance determinant elements, PAH and detoxification system hidden Markov model elements cluster by season and location. Increasing taxonomic abundance is indicated by darker blue boxes. The horizontal color bar at the top reflects sample collection location (wastewater treatment plant (top green), nearshore (top blue) and open sound (top orange)).

Overall, antibiotic resistance determinant (ARD) elements, PAH and detoxification system hidden Markov model elements cluster by season and location. Location types include wastewater treatment plant, nearshore and open sound as shown in Fig 8. The highest percentage of ARD elements (resistance genes, mobile genetic elements and transcriptional regulators) was found in WWTP samples as in our previous study. Detoxification systems including PAH degradation Pfams showed no major differences in any of the samples. Surprisingly, we detected no increase in metal resistance, detoxification or PAH associated Pfams in the Lower Duwamish River EPA superfund site sample despite a long history of anthropogenic exposure to arsenic, PAHs and other contaminants. Pfams associated with metal resistance showed the lowest abundance among all of the samples. However, when combined, ARD and detoxification system abundances reflect a subtle gradient as shown in Fig 5c similar to gradients observed in sewage and pathogenic genera detection.

## Conclusions

This is the first study to introduce longitudinal sampling of Puget Sound nearshore and open sound surface waters and wastewater treatment effluent using WMS methods. At a minimum, it helps establish baseline data in Puget Sound describing microbial community and function leading to a better understanding of descriptors of healthy versus abnormal or human-impacted ecosystems. Overall, we found a highly similar microbial community structure for geographically disparate open sound sites sampled in the same season one year apart. We also saw distinct seasonal and local sampling site variability relating to taxonomy, species diversity, predominant species, antibiotic resistance determinants and anthropogenic indicators.

Recently sequenced marine bacterial genomes provided much greater taxonomic detail at the species level compared to our previous study. Measuring seasonal variations in bacteria species which interact with microeukaryotes such as diatoms can help reveal the complex dynamics of marine microbial ecology [56].

Antibiotic resistance determinants clustered according to location. WWTP effluent samples showed the highest ARD percentage values and offshore samples the lowest. Detoxification systems showed little variation in locational or seasonal samples confirming a previous analysis highlighting the lack of adaption of nearshore or open ocean marine genomes to anthropogenic impacts [37]. However, significant differences were detected in sewage related bacterial families and pathogenic genera in WWTP, Marina and Duwamish River samples suggesting a gradient of human impact augmented by seasonal differences.

Challenges in our study included limited sampling sites, sampling frequency and consistency of sampling seasons; ideally, the same sites would each be sampled at the same time in two different seasons. Future WMS experiments will include more frequent sampling and standardization of sampling times as well as expansion of ecosystems beyond our current open sound, Marina and WWTP environments. Dramatic increases in DNA sequencing throughput now allow simultaneous capture of functional information and taxonomic information equivalent in resolution to targeted methods [57, 58]. To accommodate “big data” sequence analysis we used software developed for searching international sequence data repositories which is thousands of time faster than BLASTP or BLASTX [28]. Techniques such as single cell sequencing will continue to uncover significant marine species not amenable to conventional culturing methods and help further define taxonomic resolution. Molecular methods such as WMS and eDNA [59] may one day help identify and monitor interactions and anthropogenic impacts using a systems approach with different species resolutions, from bacteriophage to microplankton to marine vertebrates. Through an increased understanding of microbial ecology and environmental characteristics, it will be possible to identify perturbed microbial communities and potential anthropogenic sources of stress such as global warming. Overall, our results, in addition to further characterizing the Puget Sound microbial community, highlight future applications of metagenomics for environmental health monitoring and surveillance.

## Supporting information

S1 Table Puget2 project metadata for 7 sample sites. (DOCX)

S2 Table Percentages of most common phyla for 11 metagenomic samples among all detected phyla. (XLSX)

S1 Fig Puget1 and Puget2 analysis framework. (DOCX)

S2 Fig Linear regression of bacterial class abundance versus salinity for 14 Puget1 and Puget2 project samples. (DOCX)

S3 Fig Linear regression of bacterial class abundance versus salinity versus temperature for 14 Puget1 and Puget2 project samples. (DOCX)

S4 Fig DNA sequence recruitment plots for the marina site sampled in different seasons. Vertical lines represent overlapping individual DNA sequence reads mapping by genomic position to the marine bacterium, *Rhodobacterales HTCC2255*. BLASTN similarity searching reveals a 3-fold greater abundance of reads mapping in summer versus winter metagenomic samples.
(DOCX)
