# Supplementary material for: Variability in metagenomic samples from the Puget Sound: Relationship to temporal and anthropogenic impacts
Source: PLoS One. 2018 Feb 13;13(2):e0192412. doi: 10.1371/journal.pone.0192412 (PMC5811002; doi:10.1371/journal.pone.0192412)
Supplement: S1 Table — (DOCX) [file pone.0192412.s001.docx]

**Table S1. Puget2 project metadata for 7 sample sites.**
