# Supplementary figures and images for: Variability in metagenomic samples from the Puget Sound: Relationship to temporal and anthropogenic impacts
Source: PLoS One. 2018 Feb 13;13(2):e0192412. doi: 10.1371/journal.pone.0192412 (PMC5811002; doi:10.1371/journal.pone.0192412)

**
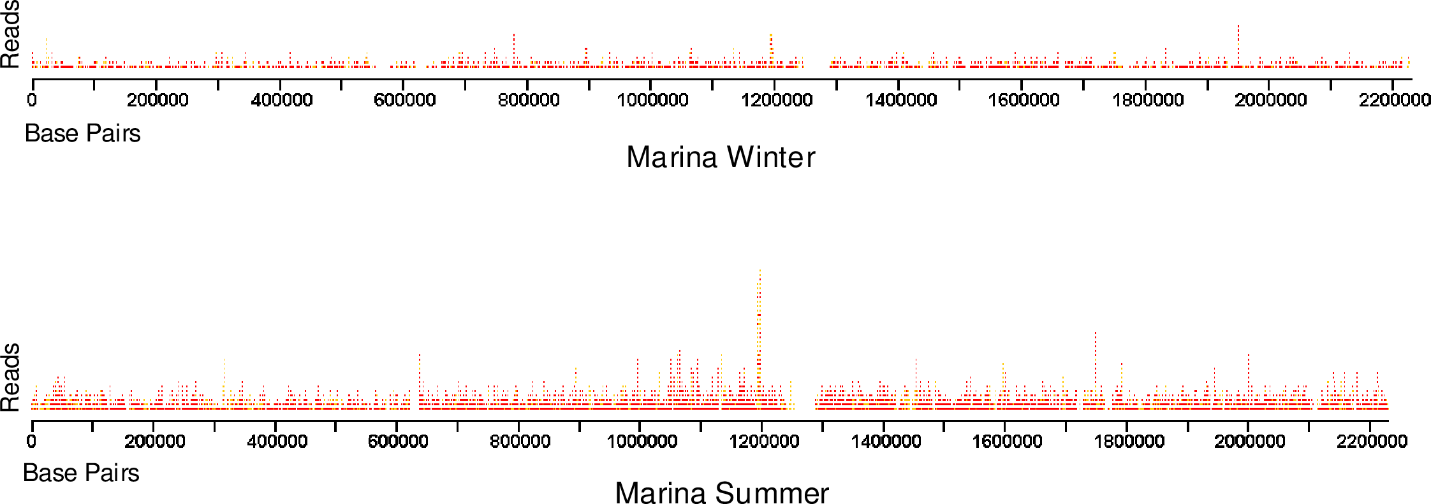
**

Supplement: S4 Fig — Vertical lines represent overlapping individual DNA sequence reads mapping by genomic position to the marine bacterium, Rhodobacterales HTCC2255. BLASTN similarity searching reveals a 3-fold greater abundance of reads mapping in summer versus winter metagenomic samples. (DOCX) [file pone.0192412.s006.docx]
