# Supplementary material for: Variability in metagenomic samples from the Puget Sound: Relationship to temporal and anthropogenic impacts
Source: PLoS One. 2018 Feb 13;13(2):e0192412. doi: 10.1371/journal.pone.0192412 (PMC5811002; doi:10.1371/journal.pone.0192412)
Supplement: Supplementary file 4 [file pone.0192412.s003.docx]

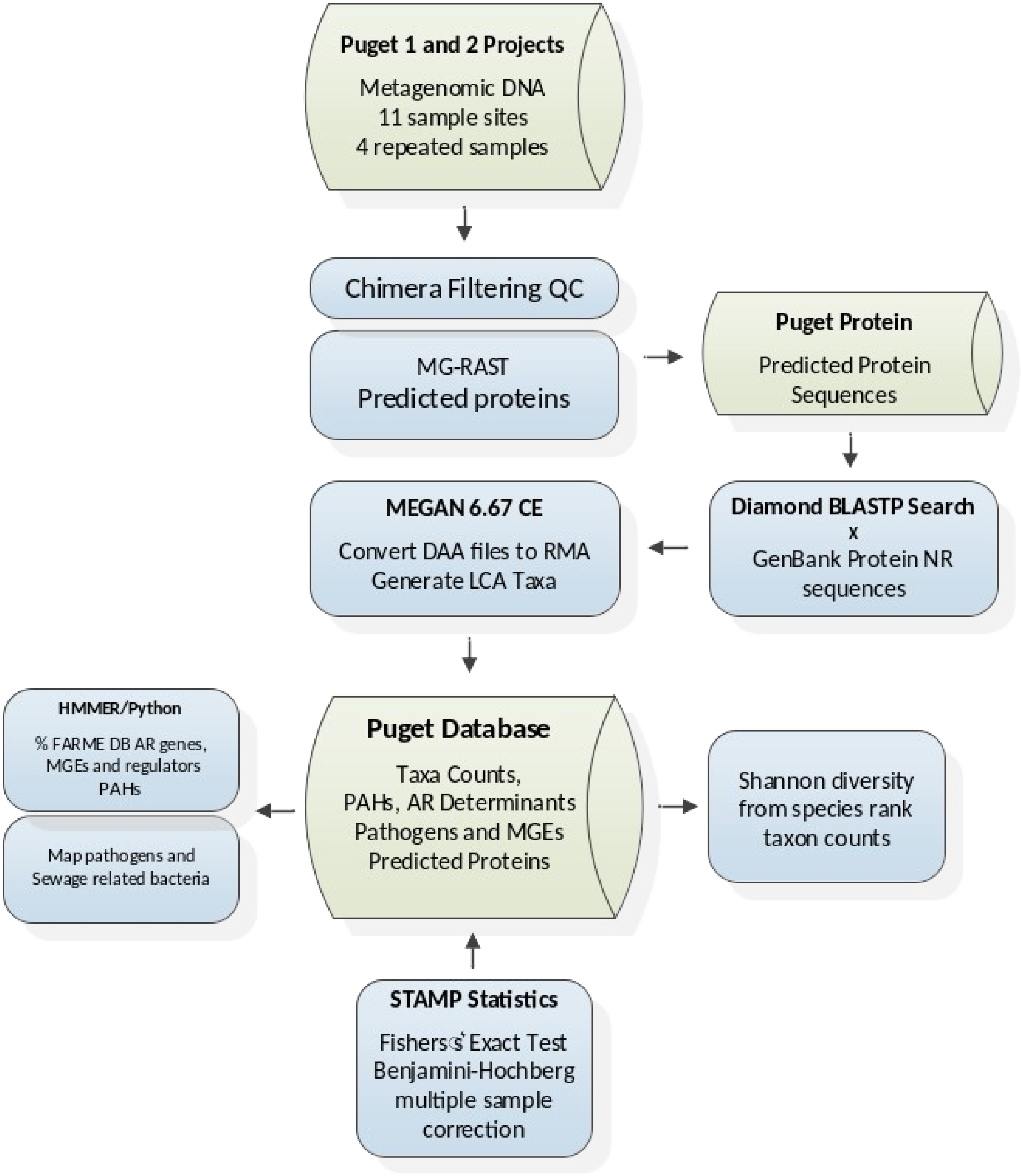
Puget 1 and 2 Projects
Metagenomic DNA
11 sample sites
4 repeated samples
Chimera Filtering QC
MG-RAST
Predicted proteins
Puget Protein
Predicted Protein
Sequences
Diamond BLASTP Search
x
GenBank Protein NR
sequences
MEGAN 6.67 CE
Convert DAA files to RMA
Generate LCA Taxa
HMMER/Python
% FARME DB AR genes,
MGEs and regulators
PAHs
Map pathogens and
Sewage related bacteria
Puget Database
Taxa Counts,
PAHs, AR Determinants
Pathogens and MGEs
Predicted Proteins
Shannon diversity
from species rank
taxon counts
STAMP Statistics
Fishers's Exact Test
Benjamini-Hochberg
multiple sample
correction
