# Supplementary material for: Variability in metagenomic samples from the Puget Sound: Relationship to temporal and anthropogenic impacts
Source: PLoS One. 2018 Feb 13;13(2):e0192412. doi: 10.1371/journal.pone.0192412 (PMC5811002; doi:10.1371/journal.pone.0192412)
Supplement: Supplementary file 5 [file pone.0192412.s004.docx]

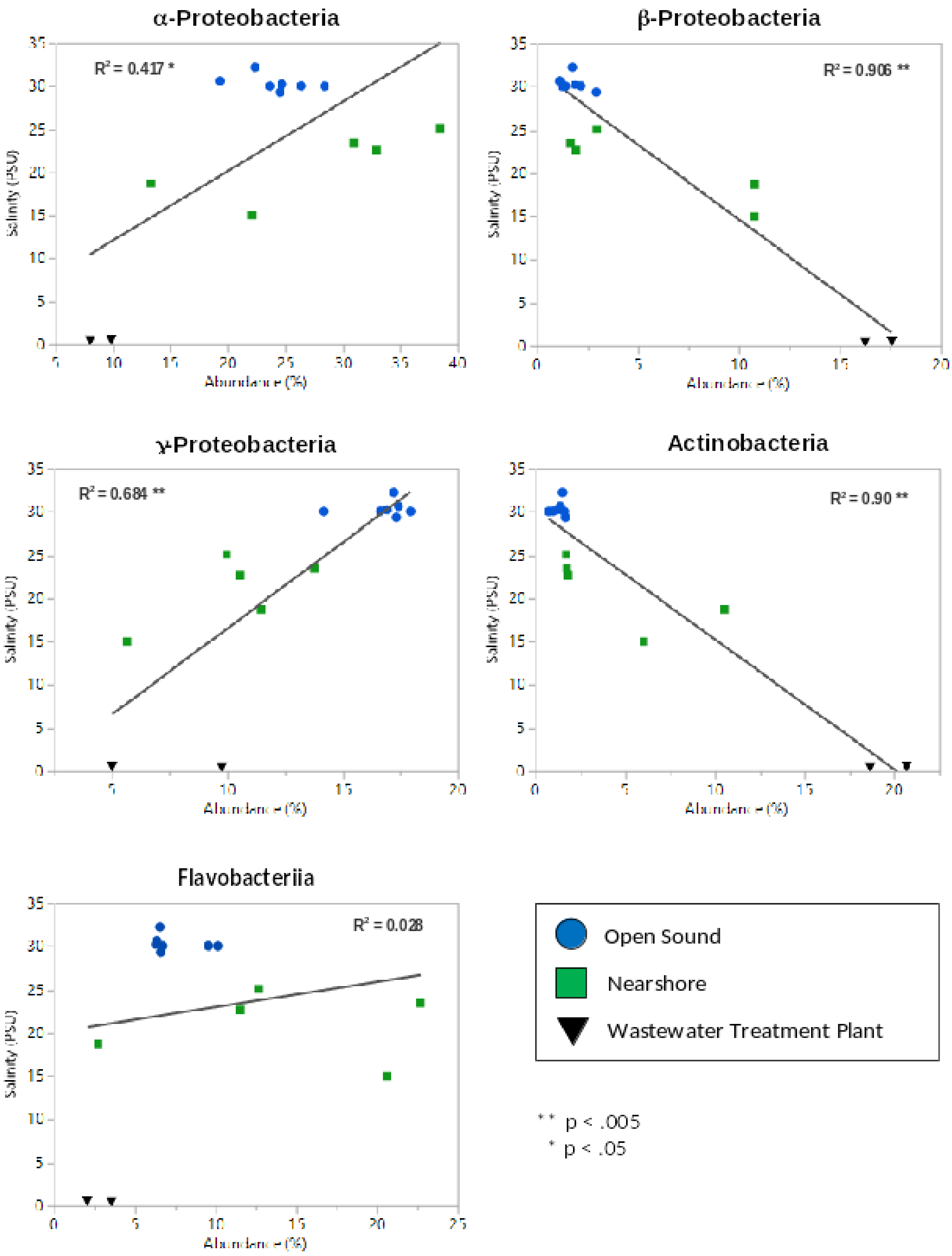
α-Proteobacteria
R² = 0.417 *
β-Proteobacteria
R² = 0.906 **
γ-Proteobacteria
R² = 0.684 **
Actinobacteria
R² = 0.90 **
Flavobacteriia
R² = 0.028
Salinity (PSU)
Abundance (%)
Open Sound
Nearshore
Wastewater Treatment Plant
** p < .005
* p < .05
