# Supplementary material for: Variability in metagenomic samples from the Puget Sound: Relationship to temporal and anthropogenic impacts
Source: PLoS One. 2018 Feb 13;13(2):e0192412. doi: 10.1371/journal.pone.0192412 (PMC5811002; doi:10.1371/journal.pone.0192412)
Supplement: Supplementary file 6 [file pone.0192412.s005.docx]

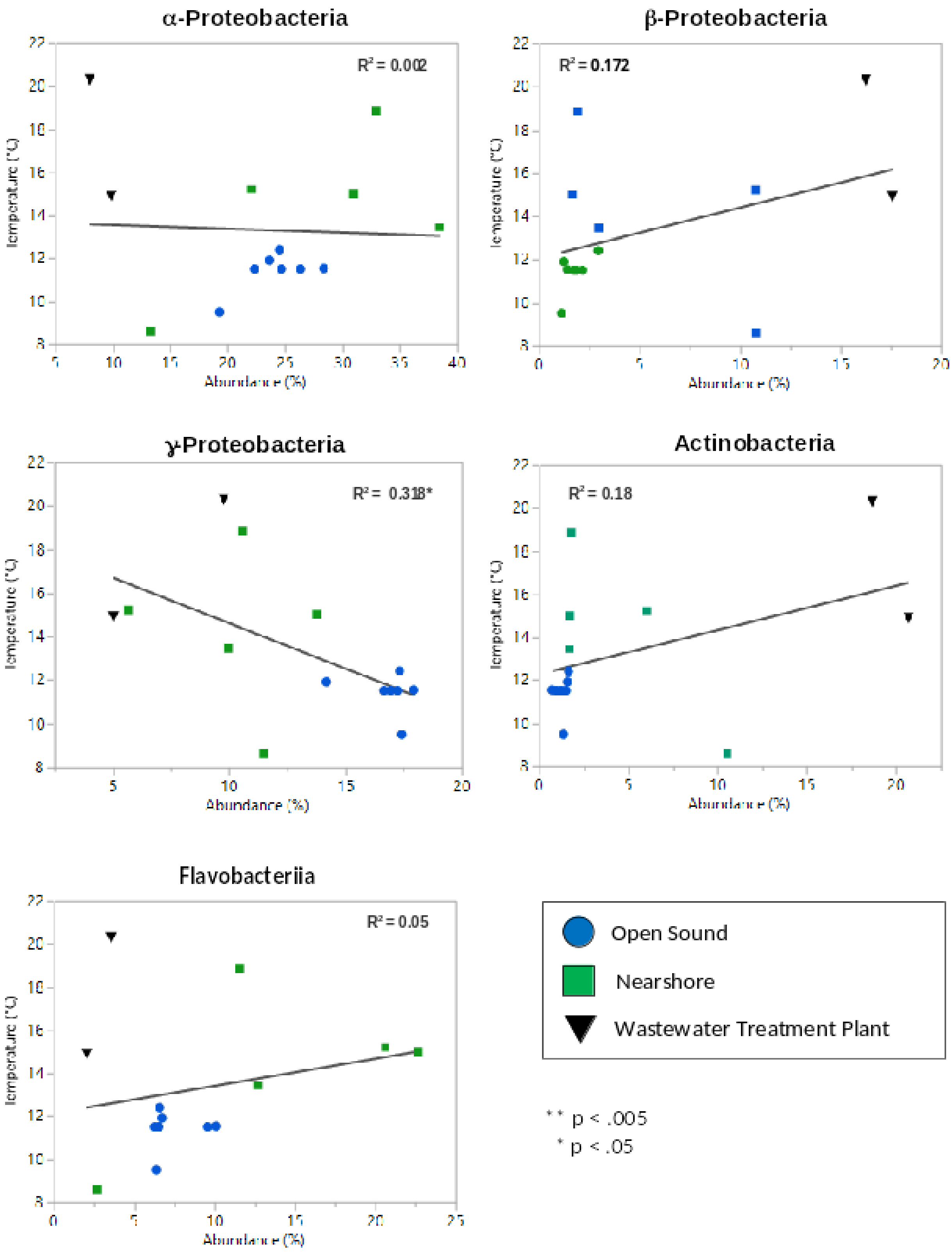

α-Proteobacteria
R² = 0.002
β-Proteobacteria
R² = 0.172
γ-Proteobacteria
R² = 0.318*
Actinobacteria
R² = 0.18
Flavobacteriia
R² = 0.05
Temperature (°C)
Abundance (%)
Open Sound
Nearshore
Wastewater Treatment Plant
** p < .005
* p < .05
